# Quantitative HBsAg and HBeAg Predict Hepatitis B Seroconversion after Initiation of HAART in HIV-HBV Coinfected Individuals

**DOI:** 10.1371/journal.pone.0061297

**Published:** 2013-04-09

**Authors:** Gail V. Matthews, Rachel J. Ali, Anchalee Avihingsanon, Janaki Amin, Rachel Hammond, Scott Bowden, Sharon R. Lewin, Joe Sasadeusz, Margaret Littlejohn, Stephen L. Locarnini, Kiat Ruxrungtham, Gregory J. Dore

**Affiliations:** 1 Kirby Institute, University of New South Wales, Sydney, Australia; 2 HIV-NAT, Thai Red Cross AIDS Research Centre, Department of Medicine, Chulongkorn University, Bangkok, Thailand; 3 Victorian Infectious Diseases Reference Laboratory, Melbourne, Australia; 4 Department of Medicine, Monash University, Melbourne, Australia; 5 Infectious Diseases Unit, Alfred Hospital, Melbourne, Australia; 6 Centre for Virology, Burnet Institute, Melbourne, Australia; 7 The Royal Melbourne Hospital, Melbourne, Australia; Duke University, United States of America

## Abstract

**Objective:**

Anti-HBe seroconversion and HBsAg loss are important therapeutic endpoints in patients with hepatitis B virus (HBV) infection. Quantitative measures of hepatitis B surface antigen (qHBsAg) and e antigen (qHBeAg) have been identified as potentially useful indicators of therapeutic response in HBV monoinfection. The aim of this study was to examine serological change including quantitative biomarkers in HIV-HBV coinfected patients initiating HBV active antiretroviral therapy (ART).

**Methods:**

HIV-HBV coinfected individuals from Thailand were followed for up to 168 weeks post ART. Rates and associations of qualitative serological change were determined. Longitudinal changes in qHBsAg and qHBeAg were measured and their utility as predictors of response examined.

**Results:**

Forty seven patients were included of whom 27 (57%) were HBeAg positive at baseline. Median CD4 count was 48 cells/mm^3^. Over a median follow-up of 108 weeks 48% (13/27) lost HBeAg, 12/27 (44%) achieved anti-HBe seroconversion and 13% (6/47) HBsAg loss. Anti-HBe seroconversion was associated with higher baseline ALT (p = 0.034), lower qHBsAg (p = 0.015), lower qHBeAg (p = 0.031) and greater HBV DNA decline to week 24 (p = 0.045). Sensitivity and specificity for qHBsAg and qHBeAg decline of >0.5 log at week 12 and >1.0 log at week 24 were high for both anti-HBe seroconversion and HBsAg loss.

**Conclusions:**

Rates of serological change in these HIV-HBV coinfected individuals with advanced immunodeficiency initiating HBV-active ART were high. Baseline and on treatment factors were identified that were associated with a greater likelihood of subsequent anti-HBe seroconversion, including both quantitative HBsAg and HBeAg, suggesting these biomarkers may have utility in this clinical setting.

## Background

Immunodeficiency induced by HIV has a significant negative impact on the natural history of hepatitis B virus (HBV) infection with greater progression to chronic HBV infection and lower rates of anti-hepatitis B e antigen (anti-HBe) seroconversion or hepatitis B surface antigen (HBsAg) loss [1]. Further, HBV DNA levels are generally higher and the risk of progression to end stage liver disease and hepatocellular carcinoma increased [2].

Although HBV virological suppression is one of the most important therapeutic goals in the treatment of HBV infection other therapeutic endpoints, such as anti-HBe seroconversion and HBsAg loss, may also be important. With nucleos(t)ide agents (NAs) however, these endpoints are generally uncommon with rates of HBsAg loss in HBV monoinfected patients of less than 10%, even with extended follow-up [3] [4]. HBV treatment serological responses may be higher in HIV-HBV coinfected populations {Piroth, 2010 #1539}, possibly related to immune restoration after ART initiation. In a recent report in lamivudine experienced Caucasian coinfected patients after 5 years of TDF-containing ART, HBeAg loss was reported at 46% and HBsAg loss in 12% [5].

Quantitative HBsAg (qHBsAg) has been described as a good correlate of viral replication since the early 1990s [6] but the lack of available commercial assays has constrained its use until recently. Several studies have shown that qHBsAg may have a role in predicting response to interferon (IFN)-based therapy in both Asian and Caucasian mono-infected populations. Baseline quantitative HBsAg (qHBsAg) has been shown to be associated with both subsequent sustained anti-HBe seroconversion and HBsAg loss. In addition, both absolute thresholds and declines in qHBsAg at early therapeutic timepoints have been identified that correlate with subsequent response with high positive and/or negative predictive values [7] [8], [9].

Very few studies have examined qHBsAg kinetics in HIV-HBV coinfected patients. One small series of French HIV-HBV coinfected patients on stable TDF-containing ART suggested HBsAg declines occurred in only around one third of patients [10]. A further French study was recently published in 143 antiretroviral experienced individuals treated with TDF [11]. In this study the rates of HBsAg and HBeAg loss over time were lower than in other studies at 4% and 21% respectively, and patients demonstrated only a slow reduction in HBsAg over time with an overall mean decline of −0.019 log_10_ IU/ml per month. Both of the above studies were performed after HAART stabilization in populations that are predominately Genotype A and Caucasian.

In this paper we prospectively examined the qualitative and quantitative serological changes at and following initiation of HBV-active highly active antiretroviral therapy (HAART) in antiretroviral naive HIV-HBV coinfected individuals in Thailand and examined the utility of these biomarkers as predictors of response in this setting.

## Methods

### Ethics statement

All subjects gave informed written consent for participation in the study and the study was approved by the relevant Human Research Ethics Committees/Institutional review boards in Thailand (Faculty of Medicine, Chulalongkorn Univeristy, Bangkok) and at the University of New South Wales, Australia. All research was conducted according to the principles expressed in the Declaration of Helsinki.

HIV-HBV coinfected antiretroviral naïve individuals were included in this analysis who initiated HBV-active ART within the context of two randomised clinical trials between August 2004 and December 2006 at the HIV-NAT clinical trials centre in Bangkok, Thailand [12], [13]. For the first 48 weeks all subjects were treated with HBV active ART containing either LAM (lamivudine), emtricitabine (FTC) or Tenofovir (TDF). Thereafter all subjects were treated with TDF containing ART and continued in observational follow-up. Patients with hepatitis C or delta were excluded. Of 54 originally randomised subjects, 47 continued follow-up for a median of 108 weeks and were eligible for inclusion in this analysis.

Serum samples were stored at weeks 4, 8, 12, 24, 36, 48 during the first 48 weeks, then every 3 months thereafter. Stored samples were retrospectively analysed to determine quantitative HBsAg and HBeAg.

### Quantification of HBsAg

Serum HBsAg titres were measured by enzyme immunoassay (EIA) using the ARCHITECT platform (Abbott Laboratories, Chicago, IL), as per the manufacturer's instructions. This quantitative assay (Abbott Diagnostics, Sligo, Ireland) is calibrated to the WHO reference standard and has a dynamic range of 0.05–250 International Units (IU)/mL. Samples were initially diluted 1 in 1000 with HBsAg Manual Diluent (Abbott Diagnostics) and were subsequently re-tested (with a lower or higher dilution) if they were below or above the assay dynamic range.

### Quantification of HBeAg

The in-house quantitative HBeAg assay used has been previously described [14]. Serum HBeAg was measured on the ARCHITECT platform using the Abbott Diagnostics HBeAg kit. An in-house working HBeAg standard was prepared from a pool of high-titre HBeAg positive specimens and calibrated against the Paul Ehrlich (PE) Institute (Langen, Germany) HBeAg reference standard (100 PE IU/mL). The working standard was then used to generate a calibration curve within the linear range of the assay (approx 0.5–100 PE IU/mL). Samples were initially diluted 1 in 10 with HBsAg Manual Diluent (Abbott Diagnostics) and were re-tested if required to fall within the assay dynamic range.

All analyses were performed with Stata 12.0, using graphical displays of data and descriptive statistics [e.g. median, interquartile range (IQR), percentages, positive predictive value]. Univariate statistical associations were assessed using Pearson chi-squared for categorical data and Kruskal-Wallis for continuous data. Statistical significance was defined using a P-value of less than 0.05.

### Quantification of HBV DNA

HBV viral load testing was performed using the Abbott RealTi*m*e HBV test (Abbott Molecular, Des Plaines, IL) according to the manufacturer's instructions with a lower limit of quantification of 20 IU/ml.

## Results

Subjects were followed for a median of 108 weeks (range, 48–168 weeks) following ART initiation. Baseline characteristics of the 47 subjects are given in Table 1.The mean age was 34 years, and 66% were male. HBV genotype was C in 83%, B in 8% and 1 patient had G. The rest were not able to be genotyped.

**Table 1:** Baseline Characteristics of study group overall by qualitative HBeAg status.

	HBeAg positive (n = 27)	HBeAg negative (n = 20)	p-value
Male gender	17 (63%)	14 (70%)	0.615
Median Age at enrolment	31	35	0.065
HBV genotype C	23 (85%)	16 (80%)	0.641
Median duration of follow up(weeks)	120	96	0.216
Median baseline HBV DNA (log_10_ IU/mL)	8.19	7.55	**0.007**
Median baseline CD4 (cells/mm^3^)	39	75	0.647
Median baseline HIV RNA (log_10_ c/mL)	4.77	4.65	0.474
Median baseline ALT (IU/L)	39	47	0.459
Median baseline qHBsAg (log_10_ IU/mL)	5	4.12	**0.002**
Median baseline qHBeAg in eAg+ (log_10_ PE IU/mL)	3.02		

Prior to ART initiation the median nadir CD4 count was 48 cells/mm^3^ (IQ range 18–190 cells/mm^3^), median HIV viral load 4.71 log_10_ c/ml (IQR: 4.34–5.17 log_10_ c/ml) and median HBV DNA was 7.86 log_10_ IU/ml (IQR: 7.06–8.25 log_10_ IU/ml). Median ALT was 39 IU/L (ULN 40 IU/L), and was normal in 53% (25/47).

Over the follow-up period complete virological suppression of HIV (<50 c/ml) and HBV (<20 IU/ml) was observed in 100% and 72% of subjects, respectively. In the subjects without complete HBV suppression, HBV DNA ranged from 30–868 IU/ml with no subject having a HBV DNA level >1000 IU/ml. CD4 count restoration was marked with a rise over follow-up in the median CD4 count of 217 cell/mm^3^.

### Qualitative serological change

#### HBeAg

**Figure 1:**
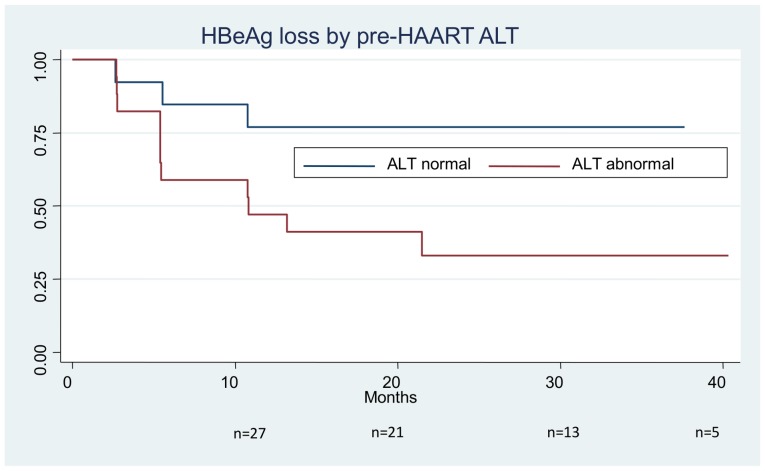
Kaplan-Meier curve of HBeAg loss after HAART initiation by pre-HAART ALT.

Prior to ART initiation 57% (27/47) of subjects were HBeAg positive. Of these subjects, 48% (13/27) lost HBeAg and 44% (12/27) achieved anti-HBe seroconversion during follow-up. Two subjects had subsequent sustained anti-HBe seroreversions, therefore the HBeAg positive proportion at last visit was 34% (16/47).Of the two seroreversions, one seroconverted for anti-HBe transiently at week eight of follow-up but remained persistently HBeAg positive from week 12 onwards and the other subject seroreverted to HBeAg positive at their final follow-up visit at week 72. A third subject seroreverted transiently to HBeAg positive at week 48 but subsequently lost HBeAg again (without seroconversion) at week 72.Kaplan-Meier analysis demonstrated the cumulative rate of HBeAg loss to be 13% at 3 months, 30% at 6 months, 40% at 12 months, 46% at 18 months, and 48% at 24 months (Figure 1). Subjects with and without anti-HBe seroconversion were compared with respect to a number of baseline and on treatment variables (Table 2). Seroconversion was more common in those with higher baseline ALT (48 IU/L versus 30 IU/L, p = 0.034) and in those with a more pronounced reduction in HBV DNA through week 24 (p = 0.045), but not with age, gender, nadir CD4 count, or baseline HBV DNA level. In particular increase in median CD4 count at week 12 and week 24 of therapy was also not associated with anti-HBe seroconversion.

**Table 2:** Characteristics of anti-HBe seroconverters compared to those with no seroconversion.

	No antiHBe seroconversion	antiHBe seroconversion	P value
	(n = 15)	(n = 12)	
Male Gender	60% (n = 9)	67% (n = 8)	0.722
Median age (years)	32.5	30	0.283
Median nadir CD4 (cells/mm^3^)	46	32	0.341
Median pre-HAART HBV DNA (log_10_ IU/ml)	8.00	8.34	0.380
Median pre HAART ALT (IU/ml)	30	48	**0.034**
Median HBV DNA decline to week 24 (log_10_ IU/ml)	6.14	7.42	**0.045**
Median gain in CD4 to week 24 (cells/mm^3^)	86	85	0.733
HBV Genotype C	10 (83%)	13 (87%)	0.809
Median BL log HIV RNA	4.68	5.06	0.435

#### HBsAg

Qualitative HBsAg loss occurred in 13% of subjects overall (n = 6), of whom 5 were HBeAg positive and one HBeAg negative at baseline. Three of 6 subjects who lost HBsAg also seroconverted for anti-HBs. HBsAg loss occurred between weeks 24 and 96 (week 24 (n = 1), week 36 (n = 1), week 48 (n = 2), week 72 (n = 1), and week 96 (n = 1)). The subject who lost HBsAg at week 96 did so only transiently, having regained HBsAg by week 120. No other seroreversions to HBsAg positive occurred.

### Quantitative serological change

Quantitative HBsAg was available from baseline and over follow-up for 46 of 47 subjects, and quantitative HBeAg for all 27 HBeAg positive subjects.

**Figure 2:**
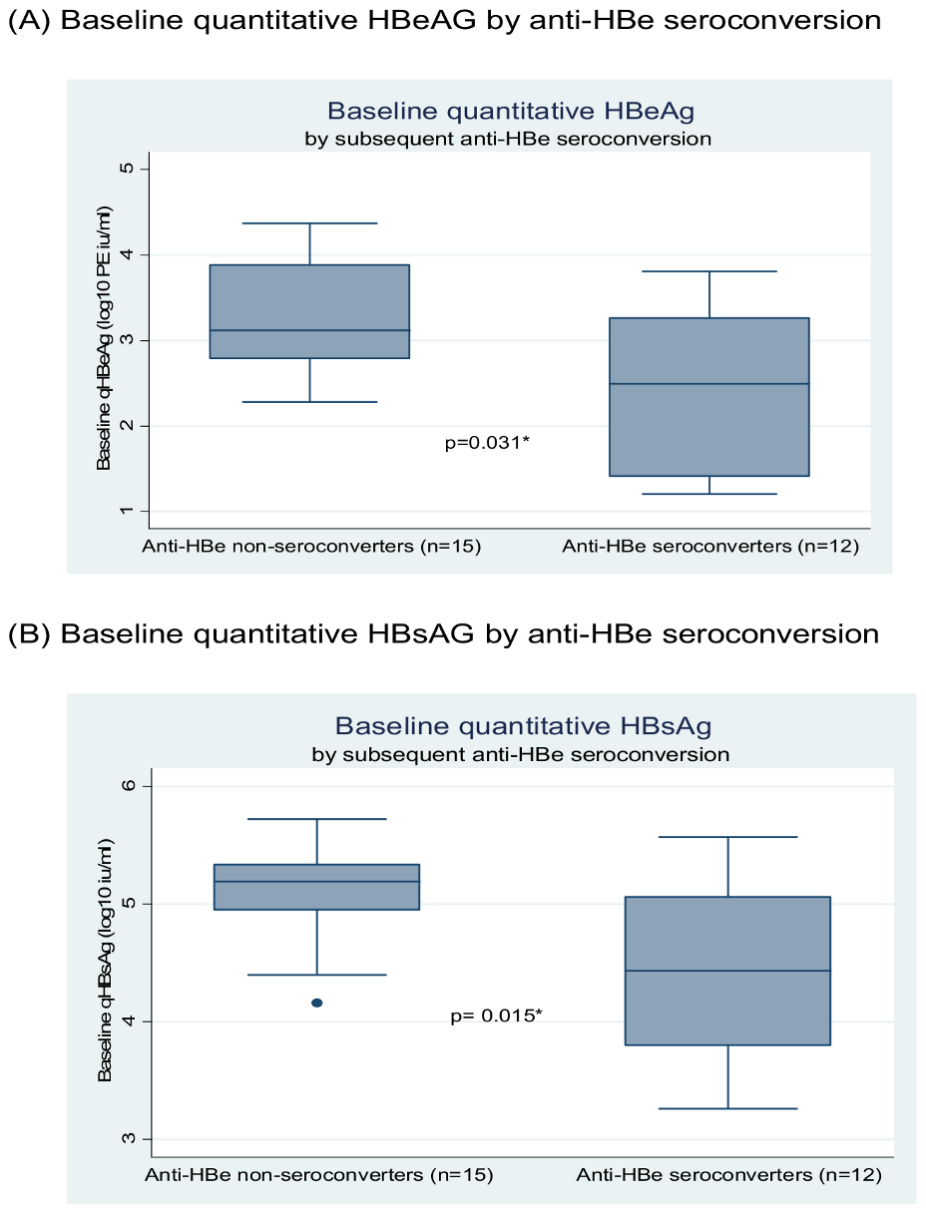
Plots of (A) Baseline quantitative HBeAg and (B) Baseline quantitative HBsAg in anti-HBe seroconverters and non-seroconverters.

In the 27 HBeAg positive subjects the median baseline quantitative HBeAg (qHBeAg) was 3.02 log_10_ PE IU/mL (range, 1.2 log_10_ PE IU/mL l–4.37 log_10_ PE IU/mL). Baseline qHBeAg correlated with baseline qHBsAg (p = 0.001) but was not associated with any other baseline variables, including gender (p = 0.188), age (p = 0.258), ALT (p = 0.276), HBV DNA (p = 0.23), HIV RNA (p = 0.216), or nadir CD4 count (p = 0.119). Baseline qHBeAg was significantly lower in those with subsequent anti-HBe seroconversion (2.5 log_10_ PE IU/mL vs 3.12 log_10_ PE IU/mL, p = 0.031) (Fig. 2).

**Figure 3:**
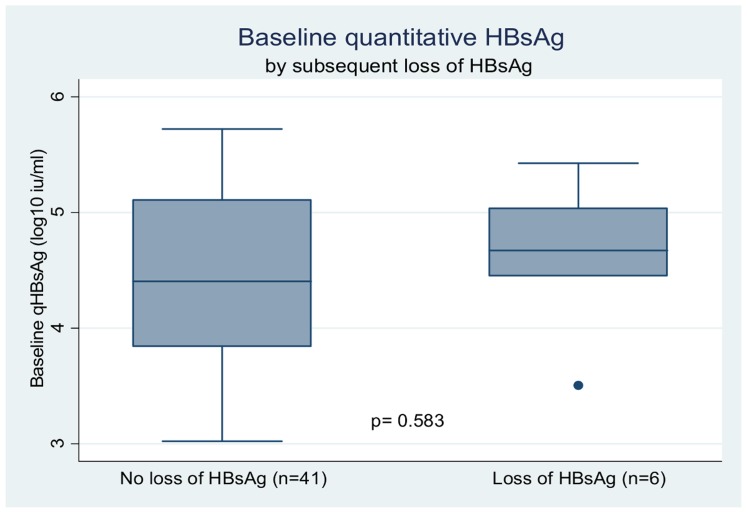
Plots of Baseline Quantitative HBsAg in those who remained HBsAg positive throughout followup and those who lost HBsAg.

Median baseline qHBsAg was 4.4log_10_ Iu/ml (IQR 3.92–5.07) and correlated with both higher baseline HBV DNA (p = 0.038) and positive HBeAg status (p = 0.002) but not CD4 count (p = 0.866) ALT (p = 0.255) or HBV genotype C (p = 0.552). While median baseline qHBsAg was not significantly different in those who subsequently lost HBsAg (4.7 log_10_ IU/mLvs 4.4 log_10_ IU/mL, p = 0.583) (Fig 3), it was significantly lower in those who subsequently seroconverted for anti-HBe (4.41 log_10_ IU/mLvs 5.25 log_10_ IU/mL, p = 0.015) (Fig 2).

### Longitudinal change in quantitative markers

Median decline of qHBeAg during follow-up was 1.37 log_10_ and was significantly greater in those who anti-HBe seroconverted (2.41 log_10_) than those who did not (0.98 log_10_, p = 0.009).

**Figure 4:**
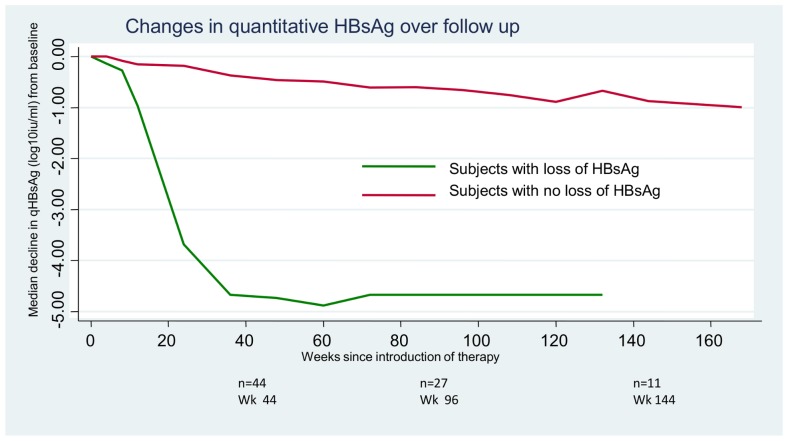
Median change in qHBsAg from baseline by loss of HBsAg.

qHBsAg declined a median of 0.75 log_10_ over follow-up and similarly this decline was significantly greater in those subjects who lost HBsAg compared to those who did not (4.67 log_10_ vs 0.65 log_10_, p = 0.001) (Fig 4). However, the qHBsAg decline was significant even in those subjects who did not lose HBsAg (p<0.001). Since follow-up was somewhat variable after week 48, qHbsAg decline was also examined as a rate. The overall rate of HBsAg decline was 0.028 IU/ml/month, 0.636 IU/ml/month in those with HBsAg loss and 0.020 IU/ml/month in those without loss (p = 0.0002). The rate of qHBsAg decline was similar over the first 48 weeks in TDF vs non-TDF containing arms (0.032 IU/ml/year vs 0.016 IU/ml/year respectively, p = 0.57), and in HBeAg positive and HBeAg negative groups (0.026 IU/ml/month vs 0.021 IU/ml/month, p = 0.50)

The sensitivity and specificity of a reduction in HBsAg at weeks 12 and 24 were examined for anti-HBe seroconversion and HBsAg loss (Table 3). The most sensitive marker of anti-HBe seroconversion was a reduction in HBsAg of >0.5 logs at week 12 (100%). Later reduction in HBsAg (>1.0 decline at week 24) was less sensitive (sens 50%). However, a >1.0 log decline at 24 weeks had high specificity (spec 92%). A >0.5 log decline in qHBeAg at week 12 was a less sensitive marker for antiHBe seroconversion (sens 58%) but the specificity for this measure was very high (spec 100%).

**Table 3:** Sensitivity and specificity values for changes in qHBsAg and qHBeAg at weeks 12 and 24.

	Week 12			Week 24	
	Se	Sp		Se	Sp
**Anti-HBe seroconversion**					
>0.5 log decline HBsAg	100 (12/12)	53 (8/15)	>1.0 log decline HBsAg	50 (6/12)	92 (12/13)
>0.5 log decline HBeAg	58 (7/12)	100 (15/15)	>0.5 log decline HBeAg	83 (10/12)	86 (13/15)
**HBsAg loss**					
>0.5 log decline HBsAg	100 (6/6)	43 (17/40)	>1.0 log decline HBsAg	100 (6/6)	84 (32/38)

Se: sensitivity; Sp: specificity

For HBsAg loss a reduction in qHBsAg of >0.5 log at week 12 or >1.0 log at week 24 was also highly sensitive (100%). However, specificity of HBsAg loss was variable, being low for >0.5 log decline at week 12 (43%) and high for >1.0 log decline at week 24 (84%).

The utility of qHBsAg decline at week 12 in predicting week 48 HBV DNA suppression was also examined. In 85% of subjects, HBV DNA was <20 IU/mL by week 48. A drop in qHBsAg of greater than 0.5 log_10_ IU/mL by week 12 was neither highly sensitive (sens 67%) or specific (spec 57%) for this measure.

## Discussion

Extended follow-up in this group of Thai HIV-HBV coinfected individuals with advanced HIV disease initiating HBV-active ART demonstrates a number of important findings. Rates of qualitative seroclearance were significantly greater than those traditionally reported in NA studies [15] [16], and 2–3 fold greater than those observed in a Caucasian genotype A antiretroviral experienced HIV-HBV cohort initiating TDF therapy[11]. In our predominantly genotype C Thai cohort, anti-HBe seroconversion occurred in 33% of subjects followed for a median of just over 2 years, and HBeAg and HBsAg loss were 46% and 13%, respectively. If the analysis is confined to those subjects with elevated ALT at baseline (the usual population for HBV therapeutic trials), the rate of HBeAg loss is extremely high at 65%. Although three patients subsequently seroreverted this was usually in the context of continued fluctuating serological change rather than hepatic flare or loss of HBV virological control as described by others {Rouphael, 2007 #1649}.

The reasons for such high rates of anti-HBe seroconversion and HBsAg loss are intriguing. In NA therapeutic studies in HBV monoinfection, rates of anti-HBe seroconversion are generally only slightly higher than without treatment, ranging from 12% [17] to 21% [15] at 1 year, and eventually rising to 35–40% with 3 or 4 years of continuous therapy [18]. HBsAg loss remains uncommon achievable in between only 0–2 % [15], [17] of people on NAs, and 8% in studies of pegylated interferon [19]. Even in the era of more potent NAs such as entecavir (ETV) and TDF, HBsAg loss is unusual, particularly among Asians. In 95 Asian patients treated with ETV no patient lost HBsAg through 24 months [20] and in two recent large scale studies of HBV monoinfected patients treated with TDF the rates of HBsAg loss were 3–8% in non-Asians [3] and 0% in Asians [4].

The rates in our study were observed in the setting of marked CD4 count restoration and almost universal viral suppression of HIV and HBV, although none of these factors, including gain in CD4 cell count, were specifically associated with seroconversion. The association with baseline transaminitis is interesting. This finding, well described previously in HBV monoinfection [21] [22], confirms that individuals in the immunoactive phase of HBV disease are those most likely to respond to therapy, although it does not explain why rates in our study were so high. A previous retrospective study in a Western cohort of 82 HIV-HBV coinfected individuals treated with LMV also identified ALT as a significant predictor of seroconversion, but additionally found an association with HIV CDC stage [23]. Our findings suggest that it is likely the combination of efficient immunorestoration after HAART, together with active anti-HBV therapy that may be driving the high rates of seroconversion in our study. Thus although our population was predominantly Asian and genotype C (factors traditionally associated with low rates of seroclearance), our rates of HBsAg loss were higher than other cohorts due to the significant degree of immune recovery observed. Since none of the other HIV-HBV cohorts have prospectively reported on individuals followed from first initiation of ART therapy, it is difficult to know how these rates would compare to a group of Caucasian individuals with similar immune recovery.

The application and utility of quantitative measures of HBsAg and HBeAg have recently undergone renewed interest in the field of HBV therapeutics. Serum HBsAg correlates with intrahepatic HBV DNA and cccDNA [14] [24] and thus acts as a potential marker of successful viral control. qHBsAg has also been shown to correlate with serum HBV DNA, as has qHBeAg [25], and both have been shown to be valuable as a potential tool in the monitoring of antiviral therapy, particularly in the setting of IFN [8], [9]. In our study baseline qHBsAg levels correlated with baseline HBV DNA and HBeAg positivity as shown by others [14]. Importantly, in our study lower qHbsAg at baseline was strongly predictive of subsequent anti-HBe seroconversion. A similar finding has been reported from the Gilead 103 study in HBV monoinfection [3] in which genotype A infection was also found to correlate with seroconversion. We did not, however, observe an association with baseline HBsAg level and subsequent HBsAg loss as was also reported in the 3 year follow-up from that study, probably due the small study population.

Not surprisingly qHBsAg declined significantly in those with HBsAg loss but also declined continuously in those without HBsAg loss, likely reflecting an ongoing reduction in intrahepatic cccDNA. The reduction in qHBsAg in individuals without seroconversion demonstrated in our study is in the same range as that reported in the Gilead 103 study in HBV monoinfection [3], although higher than in the French study conducted in experienced HIV-HBV coinfected individuals ([11], and confirms that there is a continuous sustained reduction in HBsAg over time even in the absence of seroconversion. This may be important for anticipating future HBsAg loss. At last follow up in group without sAg loss one patient had an HBsAg level at <100 IU/ml and 5 had levels <1000 IU/ml (all had levels of 4–5 logs at baseline) suggesting an ongoing significant reduction in HBsAg in these patients and potential for later HBsAg loss. Indeed, in the French study 75% of individuals with an HBsAg of <400 IU/ml on entry to their cohort subsequently lost HBsAg over time [11]. In our study higher baseline ALT, greater reduction in HBV DNA over 24 weeks, lower baseline qHBsAg and lower baseline qHBeAg were all more common in those who seroconverted as compared to those who did not. Unfortunately, the relatively small numbers involved prevent further multivariate analysis to assess the relative importance of each of these factors, which undoubtedly are correlated with each other.

Prediction of seroconversion, however, is also possible by examining dynamic changes in quantitative biomarkers. The utility of changes in qHBsAg and qHBeAg as a marker of therapeutic response have been examined in HBV monoinfected patients treated with both IFN and NA therapies. [26], [27] [28]. In our study we examined the sensitivity and specificity for changes inqHBsAg and qHBeAg for anti-HBe seroconversion and HBsAg at two early time points, week 12 and week 24 (Table 3). A week 12 HBsAg decline of >0.5 log was highly sensitive for both anti-HBe seroconversion and HBsAg loss, occurring in all individuals who reached these endpoints. However it was also observed to occur in a significant number of individuals who did not reach achieve seroconversion. Whether these individuals are more likely to experience subsequent serological change with further follow-up is unknown. Indeed by week 24 the specificity of a significant HBsAg decline had improved to 92% with all but one of the subjects with a >1 log reduction experiencing antiHBe seroconversion.

On the contrary a week 12 decline of >0.5 log in qHBeAg was highly specific for anti-HBe seroconversion but had relatively poor sensitivity. By week 24 however sensitivity had improved with little reduction in specificity. Therefore a cut-off of qHbeAg <>1.0 at week 24 may be a useful marker of anti-HBe seroconversion. This is consistent with data from other studies demonstrating the utility of qHBeAg in predicting subsequent serological change. In a study of 57 monoinfected HBeAg positive patients on ETV therapy a 1.0 log decline in qHBeAg at 6 months was highly predictive of sustained response [20], and in the French experienced HIV-HBV study an HBeAg of <10 S/Co at 12 months was also highly predictive of subsequent HBeAg loss [11]. In summary both of these biomarkers may have a role in predicting subsequent positive therapeutic outcome but the most appropriate thresholds and timepoint for assessment need to be explored and validated in larger datasets

A limitation of our study is the relatively small numbers of subjects involved compared to the large licensing HBV monoinfection studies, however this is certainly the largest and most comprehensive study of in antiretroviral naïve HIV-HBV coinfected individuals initiating ART to have examined the role of these biomarkers. In addition, it is the only detailed HIV-HBV coinfection dataset available from an Asian Genotype C population in a resource limited setting, where the future burden of HBV and HIV coinfection is likely to be significant. A further limitation is the differential use of ART during the study. For the first 48 weeks of therapy HBV active regimens were different with some subjects receiving a LAM or FTC based regimen whilst others received combination HBV active ART with TDF, before all subjects were switched to a TDF based regimen after week 48. The small numbers involved make it hard to assess any possible benefit to one regimen versus another. In the original 48 week studies numbers of seroconversions were similar across all arms and in this combined analysis qHBsAg decline was similar over the first 48 weeks in TDF containing vs non-TDF containing arms suggestingno particular benefit for one regimen and supporting the concept that the main driver of seroconversion in this setting is effective immune restoration.

In summary, this data demonstrates that in advanced HIV disease, effective restoration of HBV serological responses is possible, and in combination with continued viral suppression, this is likely to have significant impact on the future burden of liver disease in this population. Both traditional (ALT) and more novel (qHBsAg and qHBeAg) biomarkers were identified at baseline as associated with a greater subsequent likelihood of serological change. Whether quantitative biomarkers will be a useful tool, particularly in resource limited settings, by which to monitor therapy, remains to be established although increasingly longitudinal changes in qHBeAg appear to be an important marker of subsequent serological response. Of particular interest, as concern over long-term TDF toxicity continues, is whether qHBsAg levels may be used to identify specific patients in whom ceasing HBV active therapy after seroconversion is safe and in whom effective immune control can be maintained. The answers to such questions may have important implications for guidelines in resource limited countries where the burden of HIV-HBV coinfection is highest.
